# Does the Use of Chitosan Contribute to Oxalate Kidney Stone Formation?

**DOI:** 10.3390/md13010141

**Published:** 2014-12-29

**Authors:** Moacir Fernandes Queiroz, Karoline Rachel Teodosio Melo, Diego Araujo Sabry, Guilherme Lanzi Sassaki, Hugo Alexandre Oliveira Rocha

**Affiliations:** 1 Department of Biochemistry, Biosciences Centre, Federal University of Rio Grande do Norte, Salgado Filho avenue 3000, Natal, RN 59078-970, Brazil; E-Mails: moacirfqn@gmail.com (M.F.Q.); melo.krt@gmail.com (K.R.T.M.); 2 Department of Biochemistry, Biological Sciences Centre, Federal University of Parana, Coronel Francisco H. dos Santos avenue S/N, Curitiba, PR CP 19031, Brazil; E-Mails: popoh_diego@hotmail.com (D.A.S.); sassaki@ufpr.br (G.L.S.); 3 Department of Biochemistry, Molecular Biology, Federal University of São Paulo, Três de Maio street 100, São Paulo, SP 04044-020, Brazil

**Keywords:** urolithiasis, antioxidant activity, calcium oxalate monohydrate crystals, copper chelation

## Abstract

Chitosan is widely used in the biomedical field due its chemical and pharmacological properties. However, intake of chitosan results in renal tissue accumulation of chitosan and promotes an increase in calcium excretion. On the other hand, the effect of chitosan on the formation of calcium oxalate crystals (CaOx) has not been described. In this work, we evaluated the antioxidant capacity of chitosan and its interference in the formation of CaOx crystals *in vitro*. Here, the chitosan obtained commercially had its identity confirmed by nuclear magnetic resonance and infrared spectroscopy. In several tests, this chitosan showed low or no antioxidant activity. However, it also showed excellent copper-chelating activity. *In vitro*, chitosan acted as an inducer mainly of monohydrate CaOx crystal formation, which is more prevalent in patients with urolithiasis. We also observed that chitosan modifies the morphology and size of these crystals, as well as changes the surface charge of the crystals, making them even more positive, which can facilitate the interaction of these crystals with renal cells. Chitosan greatly influences the formation of crystals *in vitro*, and *in vivo* analyses should be conducted to assess the risk of using chitosan.

## 1. Introduction

Chitin and its derivative, chitosan, are biopolymers obtained from a large number of terrestrial and marine sources. They have become increasingly important since the study of fishing tailings of crustaceans began in the 1970s. Since then, various studies have been conducted on their properties, which provided a basis for the use of these two polymers in industrial and biotechnological applications [1]. Another factor that partly explains the industrial applicability of chitin/chitosan is the fact that chitin is the second most abundant natural polymer around the world. Chitin is composed of units of d-*N*-acetyl glucosamine linked together by β-(1–4). Chitosan is obtained from chitin, which undergoes different processes that remove the acetyl groups. The process of deacetylation of chitin to form chitosan is not complete, so by controlling the reaction conditions, it is possible to obtain chitosan with different levels of acetylation. Therefore, chitosan is defined as a linear polysaccharide formed by the random distribution of two monosaccharides, d-glucosamine and d-*N*-acetyl-glucosamine, which are joined together by a β-(1–4) bond. The amine groups are positively charged under physiological conditions [2].

Currently, many of the polymers used in various areas are synthetic materials, but their biocompatibility and biodegradability are very limited. On the other hand, chitosan is well known for its biocompatibility by presenting unique characteristics, such as a pH-dependent behavior, namely regarding its molecular conformation and solubility in the environment in which it is found, namely mucoadhesivity and the ease of overcoming the epithelial junctions. Furthermore, chitosan also has many pharmacological properties, making it a strong candidate to replace synthetic polymers [3,4]. In this context, in recent years, chitosan has been widely used for nanoparticle production; by the year 2012, there were approximately 10,000 published articles that had a nanotechnology and chitosan theme, accumulating more than 119,000 citations [5], which shows the considerable interest from the scientific community in the development of compounds made from chitosan. In addition, much of this interest is generated by the fact that chitosan transfers many of its properties to the nanoparticles formed from it. This leads to a question: Can the toxicity of chitosan also be transferred to nanocomposites? Existing data currently suggest that the polysaccharide chitosan is a compound with low toxicity [6,7] and that the chitosan nanoparticle toxicity would be more related to the nanoparticle size and the presence of other compounds than to the presence of chitosan [8].

Most of the chitosan-based nanocomposites, when administered to animals or patients, release chitosan into the blood stream [9]. The chitosan reaches several organs and tends to accumulate in the kidneys 8 h after oral administration [10,11], specifically in the proximal tubule cells [12]. It was also shown that the presence of chitosan in the body increases the amount of calcium excreted in urine [13]. Nevertheless, no studies have reported the toxicity of chitosan in renal tissue nor is there evidence that this caused the calcium oxalate crystal formation and consequent urolithiasis.

Urolithiasis is the formation of kidney stones, which can lead to tissue damage. This condition is a common cause of hospitalization. It is estimated that about 122 of 100,000 hospital admissions can be assigned to it. Moreover, once a patient is diagnosed with kidney stones, it is likely that the patient will develop new stones. Some studies have shown that 50%–70% of patients with a history of urolithiasis will have another crisis over the next 10 years [14].

The process of crystal formation occurs from urinary supersaturation, which provides nucleation of crystals. This event occurs when the crystal is used as a niche for similar crystals and/or macromolecules joined together, creating bigger and bigger particles. Once nucleated, the deposition of crystals in the other niche is easy and does not require such high levels of saturation as earlier in the process. The core can grow and add other crystals or organic matrix, forming the calculus, or be eliminated in the form of crystalluria [15]. After the crystallization, aggregation can occur, which describes the connection process of the crystals, resulting in the formation of agglomerate, which can precipitate [16].

Oxidative stress is another factor related to the formation of kidney stones [17]. Studies with mice that were induced to form kidney stones of calcium oxalate (CaOx) using different agents showed a decrease in plasma activities of antioxidant enzymes superoxide dismutase (SOD), catalase, glutathione peroxidase (GPx), glucose-6-phosphate dehydrogenase and glutathione S-transferase, as well as decreased plasma levels of free radical scavengers: vitamin E, vitamin C, protein thiol and reduced glutathione (GSH). It was also demonstrated that there was an increase in plasma lipid peroxidation marker molecules [18]. Data with patients who form CaOx calculus also show similar changes, indicating a positive correlation between the decrease of antioxidant defenses and the presence of oxalate/CaOx and CaOx crystal formation [19]. On the other hand, there are studies showing that antioxidant molecules can inhibit the formation of oxalate crystals in renal tissue [20,21].

As previously stated, chitosan is accumulated in renal tissue. Furthermore, it was demonstrated that the presence of chitosan in the body increases the amount of calcium excreted in urine [13], which is a primary factor in the formation of kidney stones. Given the above, this study investigated whether chitosan is able to induce the formation of CaOx crystals *in vitro* and whether it has antioxidant activity.

## 2. Results and Discussion

### 2.1. Chitosan Characterization

Chitosan was obtained commercially, and efforts were made to confirm its identity. To check whether there was contamination of the sample by impurities that could alter our results, we performed FTIR and ^1^H RMN analyses.

#### 2.1.1. Chitosan FTIR Analyses

The FTIR test was used to assess the functional groups present in the chitosan.

**Figure 1:**
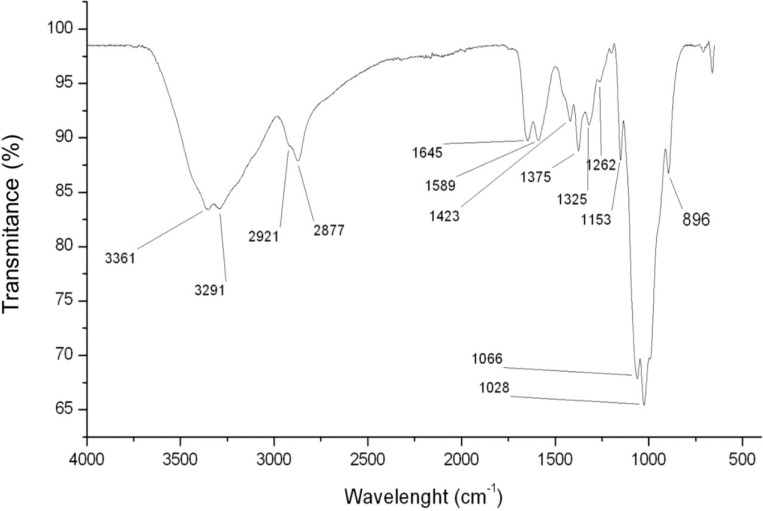
FTIR spectrum of chitosan with the characteristic signs as evidence.

In Figure 1, we can observe the infrared spectrum of chitosan. A strong band in the region 3291–361 cm^−1^ corresponds to N-H and O-H stretching, as well as the intramolecular hydrogen bonds. The absorption bands at around 2921 and 2877 cm^−1^ can be attributed to C-H symmetric and asymmetric stretching, respectively. These bands are characteristics typical of polysaccharide and are found in other polysaccharide spectra, such as xylan [22], glucans [23] and carrageenans [24]. The presence of residual *N*-acetyl groups was confirmed by the bands at around 1645 cm^−1^ (C=O stretching of amide I) and 1325 cm^−1^ (C-N stretching of amide III), respectively. We did not find the small band at 1550 cm^−1^ that corresponds to N-H bending of amide II. This is the third band characteristic of typical N-acetyl groups, and it was probably overlapped by other bands. A band at 1589 cm^−1^ corresponds to the N-H bending of the primary amine [25]. The CH_2_ bending and CH_3_ symmetrical deformations were confirmed by the presence of bands at around 1423 and 1375 cm^−1^, respectively. The absorption band at 1153 cm^−1^ can be attributed to asymmetric stretching of the C-O-C bridge. The bands at 1066 and 1028 cm^−1^ correspond to C-O stretching. All bands are found in the spectra of samples of chitosan reported by others [26,27].

Since the chitosan used in this study is from animal origin, there is always the possibility of contamination by glycosaminoglycans (GAGs), which are another type of polysaccharide found in these organisms. GAGs are sulfated, and the presence of sulfate groups covalently bonded to the polysaccharide may be confirmed in the infrared spectra by the presence of very strong bands in the region around 1260–1270 cm^−1^ [28]. In the spectrum obtained from chitosan (Figure 1), the signal at 1260 cm^−1^ is very small and, therefore, does not correspond to sulfate groups, thus ruling out contamination of chitosan by GAGs. This signal at 1260 cm^−1^ was assigned as the bending vibrations of hydroxyls present in chitosan [27]. The signal at 896 cm^−1^ corresponds to the CH bending out of the plane of the ring of monosaccharides.

#### 2.1.2. Chitosan ^1^H NMR Analyses

**Figure 2:**
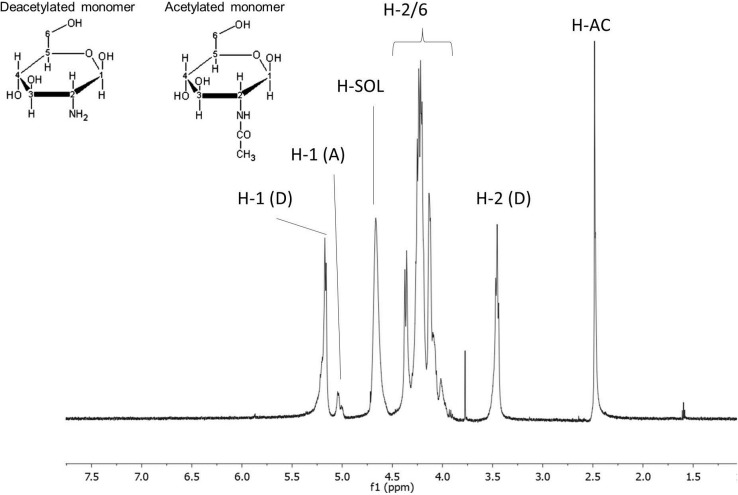
Chitosan ^1^H NMR Spectrum. AC corresponds to the acetyl group of glucosamine; D and A correspond to hydrogen of deacetylated and acetylated residues, respectively. H-SOL signaling corresponds to the solvent.

In Figure 2, we can observe the ^1^H NMR spectrum of commercial chitosan. Using this technique, we can obtain a fingerprint spectrum of each molecule. For our sample, characteristic peaks of chitosan [29,30] were identified. In the region between 3.8 and 4.2 ppm are the signals of H2, H3, H4, H5 and H6 of the aldohexoses, which overlap and, thus, make evaluation difficult. Since the signals of the anomeric protons (H1) are clear at 5.25 ppm, a peak was marked as glucosamine anomeric H (H-1 (D)) and the signal at 5 ppm was assigned as anomeric H1-N acetyl glucosamine (H-1 (A)). The peak at 3.5 ppm was assigned as H2 glucosamine (H-2 (D)), and the peak at 2.5 ppm corresponded to the hydrogens of the methyl group of *N*-acetyl-glucosamine (H-AC). The peak at 4.75 ppm corresponded to the H of the solvent.

It is also possible to discover the degree of deacetylation (DD) of chitosan through ^1^H NMR, which is an efficient method and widely accepted in the literature for this determination. In order to determine the DD, the integral of some peaks of the ^1^H NMR spectrum of chitosan was used. There are several equations for this calculation [29]. In our case, we used equations as demonstrated by Lavertu *et al.* [30] and an average DD of 76.47% ± 4.08%, which is consistent with the range indicated by the supplier (75%–85%).

### 2.2. Antioxidant Activities

Free radicals are highly reactive molecules or ions, because they have one or more unpaired electrons in their outer shell [31]. High concentrations of these radicals can generate various physiological disorders and the onset of disease [32]. To combat free radicals, organisms use various antioxidant systems formed by enzymes and/or antioxidant molecules.

Some organs, such as the liver, heart and brain, are more affected by free radicals than others, due to several factors. In the case of kidneys, free radicals cause a specific injury: the presence of CaOx crystals induces the production of reactive species, which induce the formation of more CaOx crystals, which consequently promotes the formation of more radicals and tissue damage [17]. Therefore, the use of antioxidants may prevent crystal formation and consequent renal damage [20,21].

To evaluate the antioxidant capacity of chitosan, 5 (five) tests were performed: total antioxidant capacity, reducing power, chelation of copper, iron chelation and scavenging of the hydroxyl radical.

The total antioxidant capacity test measures the ability of the electron-donating compound in an acid medium. In this test, the polysaccharide showed low activity with 1 g of sample, an activity equivalent to 30 μg of vitamin C observed.

The reducing power test also evaluates the ability of the molecule to donate electrons. This assay was expressed as the percentage activity of ascorbic acid control at 0.1 mg/mL. In this test, the chitosan (from 0.05 to 1 mg/mL) showed maximum activity at a concentration of 1 mg/mL corresponding to 34% ± 4% of the vitamin C activity. This activity was similar to those observed with other polysaccharides obtained with sulfated fucan from seaweed *Spatoglossum schröederi* and sulfated galactans obtained from *Caulerpa cupressoides*, *Caulerpa prolifera* and *Gracilaria caudata* [33]. Furthermore, the effect observed here for chitosan as a reducing agent was superior to that reported in other studies [26,34,35].

We evaluated this activity using two different methods, neither of which demonstrated chitosan’s iron-chelating activity. Several studies have shown that chitosan has iron-chelating activity, but this activity is not greater than 30% [26,34,36,37]. In addition, previous studies also reported that iron-chelating activity is dependent on both the molecular weight and the DD [36,37]. Thus, the smaller and more deacetylated the chitosan, the greater its chelating activity, as shown by Chien and colleagues [36]. These authors determined the iron-chelating activity of three different chitosans with DD of 98.5%, which possess different molecular weights (12, 95 and 318 kDa). The 95-kDa and 318-kDa chitosans (1 mg/mL) showed about 12% iron-chelating activity, whereas 12-kDa chitosan (1 mg/mL) showed about 23% iron-chelating activity. The chitosan used here has a molecular weight of 58 kDa, and its DD is about 76%, a combination of characteristics that could justify the fact that the chitosan did not exhibit iron-chelating activity.

**Figure 3:**
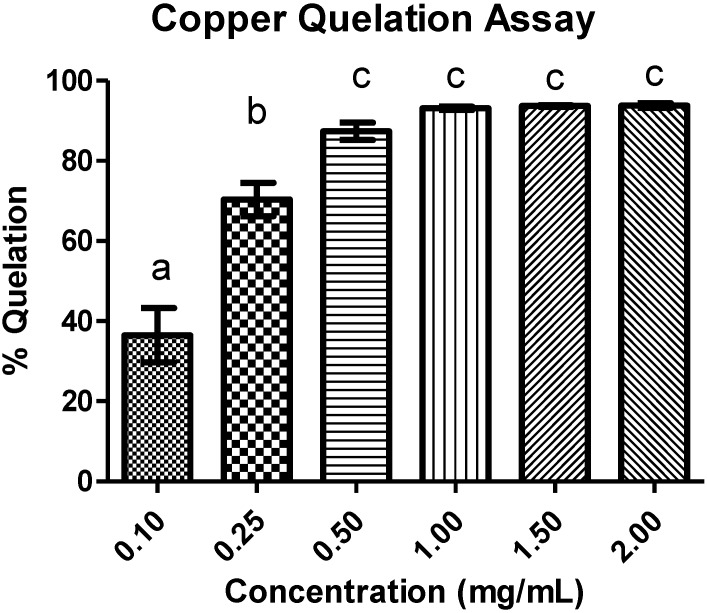
Activity of copper chelation on different chitosan concentrations. The letters indicate a significant difference between samples (*p* < 0.05).

In Figure 3, we observed the ability to chelate copper ions from chitosan; values of 90% chelation of the metal with only 0.5 mg/mL of sample were found; when this concentration was doubled, chelation of about 100% was obtained. This activity was greater than that described by Vino *et al.* [26], who showed chitosan (1 mg/mL) with a copper chelation capacity of around 88%.

Metals, such as iron and copper, in mobile environments can participate in reactions that ultimately produce hydroxyl radicals. This is very effective at causing lipid peroxidation. Therefore, the chitosan may indirectly inhibit lipid peroxidation. This property can be very important in avoiding the formation of CaOx kidney stones in that these crystals adhere to the surface of epithelial cells exactly where the surface is damaged due to lipid peroxidation [18]. The recent literature also shows that molecules with antioxidant capacity have demonstrated a potential to reduce crystal formation *in vitro* [38,39].

### 2.3. Crystal Formation

#### 2.3.1. Crystal Formation *in Vitro*

Crystal formation consists of three events: nucleation, growth and aggregation. Nucleation is the approximation of ions of different charge, forming a nucleus from which the crystals are formed. The ions present in a solution are attracted to the nucleus, forming the first nanocrystal, which will increasingly attract ions entering the phase of crystal growth. Finally, the crystals begin to collide, merge and reach a size at which precipitation occurs by setting the stage for aggregation [40].

**Figure 4:**
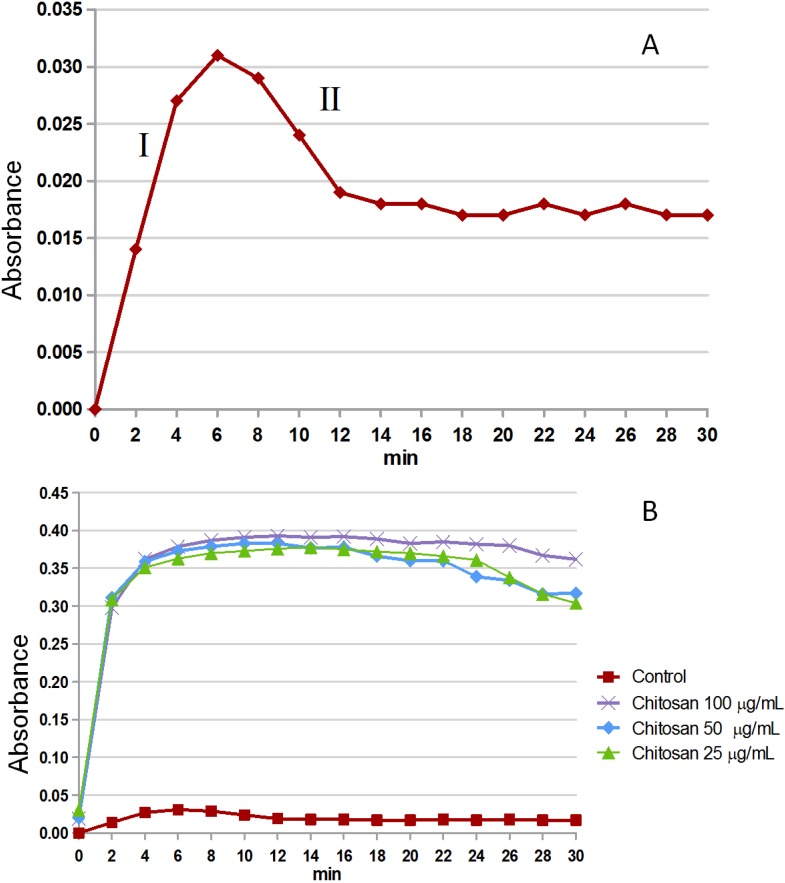
Profile of crystals forming from solutions. (**A**) Profile of the control solution formation, aggregation phases (I) and precipitation (II) are indicated with Roman numerals; (**B**) We can see the profile of crystal formation in the presence of different concentrations of chitosan.

The test of crystal formation *in vitro* evaluates the ability of the sample to inhibit or stimulate the formation of CaOx crystals. In Figure 4a, we observe the profile of CaOx crystal formation (control). The ascending portion of the curve (I) corresponds to the phases of nucleation and crystal growth, while the descending portion (II) corresponds to the aggregation/precipitation stage. When the formation of CaOx crystals was carried out in the presence of chitosan in three different concentrations (100, 50, 25 μg/mL), this revealed a significant increase in absorbance (Figure 4b). The chitosan in all conditions showed the increased CaOx nucleation/growth to be about 1500% (15-times). It was not possible to determine how the presence of chitosan interferes with the aggregation/precipitation, because after 30 min, it was still not possible to observe the precipitation of crystals.

#### 2.3.2. Crystal Morphology

The data so far show that chitosan stimulates the formation of CaOx crystals. However, we cannot say what type of crystal is being formed. Oxalate crystals morphologically differentiate into three types: monohydrate (COM), having a rectangular geometry; dihydrate (COD), present in the microscope as pyramidal or vane shapes; and trihydrate (COT), which has a complex geometry, characterized by the presence of several edges. Green and Ratan [41] showed that the COD-type crystals are found in abundance in the urine of healthy patients, and the COM type are those most commonly found in patients with urolithiasis and with a greater ability to cause cell damage. The crystals of the COD type are formed spontaneously in urine, which has different mechanisms to increase the stability of the COD crystals, which are more readily excreted. In a patient with urolithiasis, these stabilizing mechanisms are not efficient, making the COD crystals, which are not very stable, dissociate into the COM-type, which is more harmful [41]. In the renal tubule, COM crystals, positively charged, interact with cell surfaces that act as negatively charged surface. This difference in electrical charge mediates the adhesion of crystal cells. Once on the cell surface, COM crystals can reach the intracellular environment by endocytosis or other mechanisms, and when this occurs, this creates oxidative stress that promotes the release of pro-inflammatory damage and cell death [42,43].

**Figure 5:**
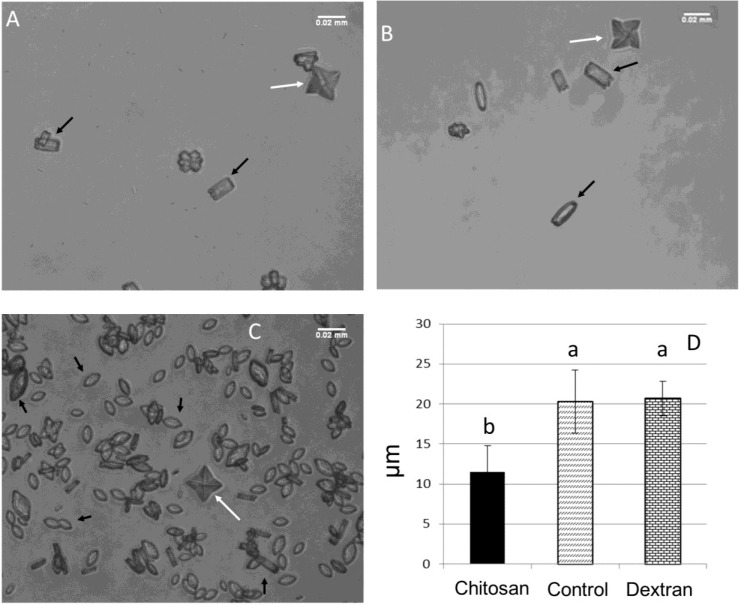
Crystal morphology analysis: comparison of the morphology of the crystals increased by 600×. (**A**) Control, with few crystals per field; (**B**) crystals formed in the presence of 100 μg/mL dextran; (**C**) crystals formed in the presence of 100 μg/mL chitosan, where we can see a large increase in the total number of crystals. White arrows indicate COD (dihydrate CaOx) and black arrows point to COM (monohydrate CaOx); (**D**) Average size with crystals formed in the presence and absence of chitosan and dextran.

In Figure 5, we can observe the crystals formed in CaOx control conditions (Figure 5a), in the presence of dextran (Figure 5b) and chitosan (Figure 5c). Comparing the three figures, one can clearly see that the number of crystals in Figure 5c is much greater. In fact, without the presence of chitosan (Figure 5a,b), an average of 9 ± 0.7 per field can be observed, whereas in the presence of chitosan, the number of crystals is increased to about 167 ± 23 per field. In Figure 5b,d, the morphology and size of the crystals were not changed by dextran. These data indicate that crystal formation in the presence of chitosan is specifically chitosan related and is not an unspecific colloidal effect.

These data confirmed what had been observed in the previous section and indicated that chitosan stimulates the formation of oxalate crystals. Another important factor that can be highlighted is that when these crystals were counted, it was found that in the control conditions, the ratio of COD:COM was 1:4 by field. The presence of chitosan modified the COD:COM ratio to 1.2:166 per field.

Not only was the number of COM crystals altered by the presence of chitosan, but the size and morphology of these crystals were also changed. The crystals in the control group exhibited a rectangular morphology (Figure 5a) and an average particle size of 20.32 ± 3.95 μM (Figure 5c). In the presence of chitosan, the COM crystals had a more spheroid shape (Figure 5b), indicating that chitosan affects the crystal lattice point, preventing the formation of perfect CaOx crystals. Furthermore, with the presence of chitosan, the average size of the crystals was 11.53 ± 3.23 μM (Figure 5c).

#### 2.3.3. Zeta Potential (ζ)

The crystals are formed spontaneously from the nearest compatible ions and have a residual charge. This charge allows them to attract or repel, thus interfering with crystal growth [44]. COM crystals are different: the positive side is rich in Ca^2+^, and the other side is rich in oxalate, which is negative. Recently, we have shown that negatively-charged polysaccharides (fucans and sulfated glucans) can associate with the positive faces of the CaOx crystals, thus reducing the surface charge of the crystals and, therefore, being able to alter the morphology of the crystals, as well as to decrease the size of the COM crystals [38]. Our question was whether the chitosan would also be associated with the surface of COM crystals. In order to answer this question, we measured the zeta potential of the crystals.

**Figure 6:**
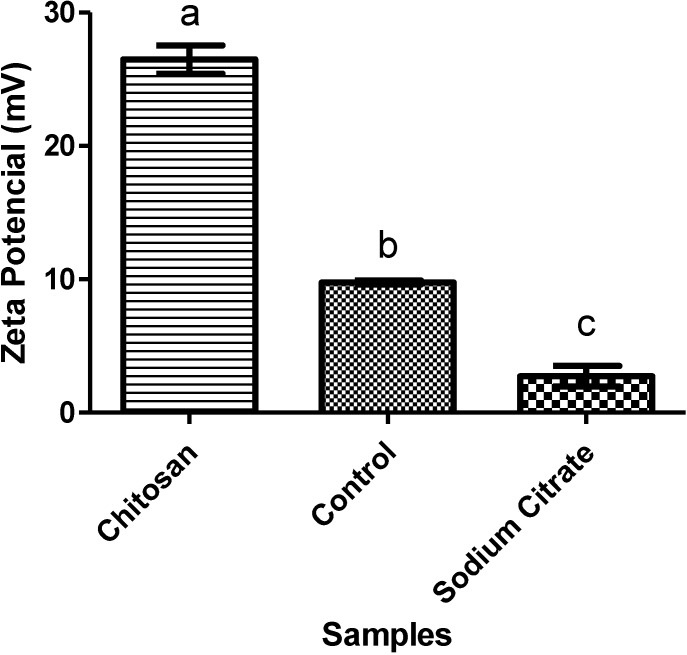
Zeta potential of the CaOx crystal samples without (control) or with chitosan or sodium citrate. Each letter indicates a statistically different group (*p* < 0.05).

The zeta potential (ζ) reflects the surface charge of particulate matter in relation to the load of the solution in which it lies. In Figure 6, we can see the total load of CaOx crystals in the presence or absence (control) of chitosan and citrate. It was observed that the crystals of the control group showed a positive ζ due to the presence of Ca^2+^. When citrate, an inhibitor of CaOx crystals, was present, the ζ decreased, due to the negative charges of citrate. Nevertheless, the opposite effect was observed in the presence of chitosan; the ζ crystals increased nearly 2.5-fold compared to ζ presented by the crystals in the control group. This increased ζ indicates that the crests of chitosan are associated with the crystals formed, since chitosan has a positive charge and is therefore able to increase the ζ CaOx crystals.

The data presented here leads us to suggest that the formation of CaOx crystals in the presence of chitosan starts with nucleation, but during the following process, chitosan starts to associate itself with the surface of the growing crystal, interfering with the crystal morphology. Furthermore, due to its positive character, chitosan by electrostatic repulsion prevents ions of Ca^2+^ from binding to the growing crystal until it reaches a point that the crystal stops growing. The calcium that does not react remains available for new crystals to be formed, which would explain the greater number of crystals formed in the presence of this polysaccharide in comparison to the control. It is worth noting that the presence of chitosan on the surface of the crystals, also by electrostatic repulsion, prevents the aggregation phase from occurring, which explains the smaller size of the crystals formed in the presence of chitosan compared to the control, as well as the fact that we did not identify the stage of aggregation/precipitation after 30 min (Section 2.3.1).

It has been shown that the presence of adverse polymers, including polysaccharides, decreases ζ crystal formation and increases the stability of COD crystals, preventing these from transforming into COM crystals [38,45]. However, with the presence of chitosan, the proportion of COM crystals, which are more damaging, compared to the COD, increased, which indicates that the chitosan was not able to stabilize the COD crystals.

Chitosan is gaining more acceptance in daily activities, since it is generally known to be a molecule without toxic effects, and its uses range from food supplement for weight loss [10,46] to even raw material for the production of nanoparticles and hydrogel drug carriers [8,47]. In 2013 alone, more than 350 articles on the use of chitosan nanoparticles as drug and/or carriers of drugs were published. Therefore, understanding the possible toxic effects of this molecule is needed. What we found here was that the literature has examples that show that chitosan has an affinity for renal tissue [10,11] and that it increases the concentration of urinary calcium [13]. Furthermore, our data show that chitosan increases the formation of CaOx crystals *in vitro*, mainly COM crystals, and it also increases the positive character of COM crystals, leaving them, in theory, more likely to interact with the negative charges on the cell surface and, thus, more likely to cause damage and urolithiasis. Therefore, we conclude that chitosan may be an inducer of renal stone formation. However, it is still too early to say for certain, because *in vivo* tests are needed to confirm the data obtained here *in vitro*. Such *in vivo* tests are currently in progress.

## 3. Experimental

### 3.1. Chitosan

The chitosan was purchased from Sigma-Aldrich (Ref: 448869–250 g; Lot: 61496MJ, St. Louis, MO, USA).

### 3.2. Fourier Transformed Infrared Spectroscopy (FTIR)

The chitosan (5 mg) was thoroughly mixed with dry potassium bromide. The infrared spectra between 500 and 4000 cm^−1^ were obtained with a tablet containing KBr and chitosan using a Thermo Nicolet Nexus 470 ESP FTIR spectrometer (Thermo Nicolet, Madison, WI, USA). Thirty-two scans at a resolution of 4 cm^−1^ were evaluated and referenced against air.

### 3.3. Nuclear Magnetic Resonance (NMR) Spectroscopy

The chitosan (50 mg) was dissolved in 800 μL of deuterium oxide (D_2_O). NMR spectra (^1^H) were obtained in a Bruker Avance III 400 MHz spectrometer (Bruker BioSpin Corporation, Billerica, MA, USA) with an inverse 5-mm broadband probe head (BBI) at 70 °C. The chemical shifts were expressed in δ relative to acetone at δ 2.21, based on sodium 2,2-dimethyl-2-silapentane-3,3,4,4,5,5-*d*_6_-5-sulfonate (DSS) at δ = 0.00 in accordance with IUPAC recommendations.

#### Determination of Deacetylation Degree

For determining the DD sample, we used the integrals of the peaks observed in the anomeric region of ^1^H NMR as demonstrated by Lavertu *et al.* [30], which applied to our chitosan. The formula used was:

(1) $$DD(\%)=\left(\frac{H1D}{H1D+H1A}\right)\times 100$$

### 3.4. Determination of Chitosan Molecular Weight

The molecular weight of chitosan was determined by high performance size exclusion chromatography (HPSEC) (GE Healthcare Bio-Sciences, Pittsburgh, PA, USA) on TSK-Gel^®^ 3000 (30 cm × 0.75 cm), with a column temperature of 60 °C. The chitosan was eluted with 0.2 M NaCl in 0.05 M acetate buffer, pH 5.0, at a flow rate of 1.0 mL/min and detected by a refractive index detector. The column was calibrated using different dextrans (10; 47; 74; 147 kDa) purchased from Sigma (St. Louis, MO, USA).

### 3.5. Antioxidant Activity

#### 3.5.1. Determination of Total Antioxidant Capacity

This assay is based on the reduction of Mo (VI) Mo (V) by chitosan and subsequent formation of a phosphate green complex/Mo (V) with acid pH. Tubes containing chitosan and reagent solution (0.6 M sulfuric acid, 28 mM sodium phosphate and 4 mM ammonium molybdate) were incubated at 95 °C for 90 min. After the mixture had cooled to room temperature, the absorbance of each solution was measured at 695 nm against a blank. Total antioxidant capacity was expressed as ascorbic acid equivalent.

#### 3.5.2. Reducing Power

The reducing power was quantified according to the methodology described by Costa *et al.* [33]. The test samples (4 mL) in different concentrations (0.25–1 mg/mL) were mixed in a phosphate buffer (0.2 M, pH 6.6) with potassium ferricyanide (1%) and incubated for 20 min at 50 °C. The reaction was interrupted by the addition of TCA (trichloroacetic acid) to 10%. Subsequently, distilled water and ferric chloride (0.1%) were added to the samples. Readings were taken at 700 nm. The data were expressed as a percentage of the activity shown by 0.1 mg/mL of vitamin C, which corresponds to 100%.

#### 3.5.3. Hydroxyl Radical Scavenging Activity Assay

The scavenging assay of the hydroxyl radical was based on the Fenton reaction (Fe^2+^ + H_2_O_2_ → Fe^3+^ + OH^−^ + OH). The results were expressed as inhibition rates. The hydroxyl radicals were generated using 3 mL of sodium phosphate buffer (150 mM, pH 7.4) containing 10 mM FeSO_4_·7H_2_O, 10 mM EDTA, 2 mM of sodium salicylate 30% H_2_O_2_ (200 mL) and different concentrations of chitosan. In the control, sodium phosphate buffer replaced H_2_O_2_. The solutions were incubated at 37 °C for 1 h, and the presence of the hydroxyl radical was detected through the monitoring of the absorbance at 510 nm. Gallic acid was used as a positive control.

#### 3.5.4. Ferrous Chelating

Both methods used the ferrozine and FeCl_2_ complex to determine the antioxidant capacity.

In the first method, chitosan at different concentrations (0.01–2 mg/mL) was added to a reaction mixture containing FeCl_2_ (0.05 mL, 2 mM) and ferrozine (0.2 mL, 5 mM). The mixture was stirred and incubated for 10 min at room temperature, and the absorbance of the mixture was measured at 562 nm against a blank. EDTA was used as standard.

In the second method, chitosan at different concentrations (0.5–2 mg/mL) was mixed with 3.7 mL of methanol and 0.01 mL of 2 mM FeCl_2_, then 0.2 mL of 5 mM ferrozine were added to initiate the reaction. The mixture was shaken vigorously and kept at room temperature for 10 min. Absorbance was determined at 562 nm against a blank, and EDTA was used as the standard.

For both methods, the chelating effect was calculated using the corresponding absorbance (A) in the formula given below, where control is the condition in the absence of chelating agents:

(2) $$Chelating\;effect(\%)=\left(\frac{A_{control}-A_{sample}}{A_{control}}\right)\times 100$$

#### 3.5.5. Copper Chelating

The ability to chelate the copper ion from the extracts was determined by the method described by Anton [48]. Pyrocatechol violet, the reagent used in this assay, has the ability to associate with certain cations, such as aluminum, copper, bismuth and thorium. In the presence of chelating agents, this combination is not formed, resulting in decreased staining. The test is performed in 96-well microplates with a reaction mixture containing different concentrations of samples (0.1–2 mg/mL), pyrocatechol violet (4 mM) and copper II sulfate pentahydrate (50 mg/mL). All wells were homogenized with the aid of a micropipette, and the solution absorbance was measured at 632 nm. The ability of the samples to chelate the copper ion was calculated using the corresponding absorbance (A) in the following formula, where control is the condition in the absence of chelating agents:

(3) $$Copper\;chelation(\%)=\left(\frac{A_{control}-A_{sample}}{A_{control}}\right)\times 100$$

### 3.6. Calcium Oxalate Crystallization Assay

The effect of polysaccharide in the crystallization of calcium oxalate was spectrophotometrically measured for 30 min at 620 nm, as described by Zhang *et al.* [39]. This assay is based on quantification by the optical density of metastable solutions of Ca^2+^ and oxalate, by means of a mixture of calcium chloride (8 mmol/L) and sodium oxalate (1 mmol/L), 200 mmol/L of sodium chloride and 10 mmol/L of sodium acetate. The concentrations of compounds present in this mixture are close to the physiological urinary concentrations. The CaCl_2_ (1.0 mmol/L) solution was constantly stirred at 37 °C, either in the absence or the presence of different concentrations of chitosan (100, 50 and 25 μg/mL). After obtaining a stable baseline, crystallization was induced by the addition of a solution of Na_2_C_2_O_4_ (1.0 mmol/L) to achieve final concentrations of 4 mmol/L of calcium and 0.5 mmol/L of oxalate. The data is presented as absorbance × time (min).

### 3.7. Image Analysis Crystal Morphology

The crystals were induced to take shape in the presence or the absence of chitosan or dextran (100 μg/mL). After 30 min, the solutions were centrifuged (5000× *g*), and the supernatant was discarded. The crystals were then suspended in 0.5 mL of water and a part of 0.1 mL was put on a histological blade and taken to a microscope. The crystal morphology was analyzed in 10 randomly selected fields at 60× magnification. Images were captured from different fields. Three different experiments were performed.

### 3.8. Zeta Potential (ζ) Measurements

The crystals were induced to form in the presence or absence of chitosan or sodium citrate (0.25 mM). After 30 min, the solutions were centrifuged (5000× *g*). The crystal concentrate was then suspended in 1.5 mL of water, and the zeta potential of the ζ samples was obtained using a Zeta Plus^®^ analyzer (Brookhaven instruments, Holtsville, NY, USA).

### 3.9. Statistical Analysis

All of the data are expressed as the mean ± standard deviation (*n* = 3). To test the difference between results, the ANOVA test was performed. The Student–Newman–Keuls test (*p* < 0.05) was used to solve similarities found by the ANOVA. All tests were performed in GraphPad Prism 5 (GraphPad Softwares, La Jolla, CA, USA).

## 4. Conclusions

The ^1^H NMR and FTIR data show that the commercial chitosan exhibited the characteristic signals of the polymer.

The commercial chitosan sigma showed low or no antioxidant activity for TAC, reducing power, hydroxyl radical scavenge and iron chelation tests; on the other hand, it showed excellent activity in the chelation of copper, reaching 100% in a concentration of 1 mg/mL.

The presence of chitosan increased the number of CaOx crystals formed, especially COM crystals, without affecting the number of COD crystals. This polymer also changed the size and morphology of crystals formed, which, due to the presence of chitosan, became more rounded. Chitosan also changes the surface charge of the crystals, making them much more positive.
